# Hypoglycemic noncardiogenic pulmonary edema: a case report

**DOI:** 10.1186/s13256-025-05413-w

**Published:** 2025-09-29

**Authors:** Filmon Tesfay, Goitom Hagos, Lidya Musie

**Affiliations:** 1 Teseney Hospital, Teseney, Eritrea; 2 Head Department of Internal Medicine, Orotta College of Medicine and Health Sciences, Asmara, Eritrea; 3 Afabet Hospital, Afabet, Eritrea

**Keywords:** Hypoglycemia, Neurogenic pulmonary edema, Noncardiogenic pulmonary edema, Case report

## Abstract

**Background:**

Hypoglycemic pulmonary edema was well addressed in the mid-1900s, and experimental data in addition to clinical reports have confirmed the association. The pathophysiology is similar to a neurogenic type of noncardiogenic pulmonary edema. Despite this, medical textbooks have not included hypoglycemia as a possible cause of this type of pulmonary edema, and only a few case reports are published, meaning that this association is often forgotten. To the best of our knowledge, our case is the first case report in over 15 years.

**Case presentation:**

A 55-year-old Eritrean male patient with known diabetes presented to our emergency room after he became unconscious at home. He was accompanied by his family. On examination, his random blood sugar was measured at 58 mg/dL, and he had a Glasgow Coma Scale score of 9/15. He was severely hypoxic, with bilateral crackles. Upon management of hypoglycemia with dextrose infusion, he improved steadily with supportive oxygen until his Glasgow Coma Scale score reached 15/15, when his pulmonary edema showed marked improvement. He was discharged, with his condition improved, on third day of admission.

**Conclusion:**

The unique presentation of pulmonary edema in a 55-year-old patient with diabetes underscores the possibility of hypoglycemia causing noncardiogenic pulmonary edema. This case contributes to the understanding of atypical complications of hypoglycemic neurologic insult.

## Background

Hypoglycemia is a restricting factor in the management of diabetes glycemia [1]. Pulmonary edema, as a complication of hypoglycemia, was well known from the 1930s to the early 1950s, as it was an acceptable practice to induce transient hypoglycemic coma as a treatment for schizophrenia [2, 3]. During those days, this association was addressed. However, the association of hypoglycemia and pulmonary edema is often forgotten, as only a few case reports are published, and there is no mention of the association in medical textbooks [4–6]. Even experimental data have confirmed the association, backing the clinical reports [7, 8]. Nielson *et al*. reported that the incidence of pulmonary edema was 3 cases in 1200 hypoglycemic coma treatments (0.25%) [2]. Death owing to acute pulmonary edema accounted for 15.9% following insulin shock treatment, as per the case series [9].

The mechanism of pulmonary edema in hypoglycemia is considered to be a neurogenic type of noncardiogenic pulmonary edema (NCPE) [3–6, 10]. The pathophysiology of hypoglycemia causing pulmonary edema is similar to neurogenic pulmonary edema, as hypoglycemia causing cerebral damage can be as severe as that caused by vascular or traumatic injury. Thus, hypoglycemic pulmonary edema is a type of neurogenic pulmonary edema. The association can even be inferred from animal studies [4, 6–8]. Neurogenic pulmonary edema (NPE) is characterized by presentation within minutes to hours following severe central nervous system insult. The most common symptom is dyspnea. Tachypnea, tachycardia, and basilar rales can be revealed from physical examination. The typical chest radiograph shows a normal heart size with bilateral alveolar opacities [11, 12]. Most cases of neurogenic pulmonary edema resolve within 48–72 hours [13]. Here, we are reporting a very rare occurrence that can contribute to the understanding of an atypical complication of hypoglycemia, in which a patient with known diabetes presented with noncardiogenic pulmonary edema after hypoglycemic neurologic insult, from which he fully recovered.

## Case presentation

**Fig. 1:**
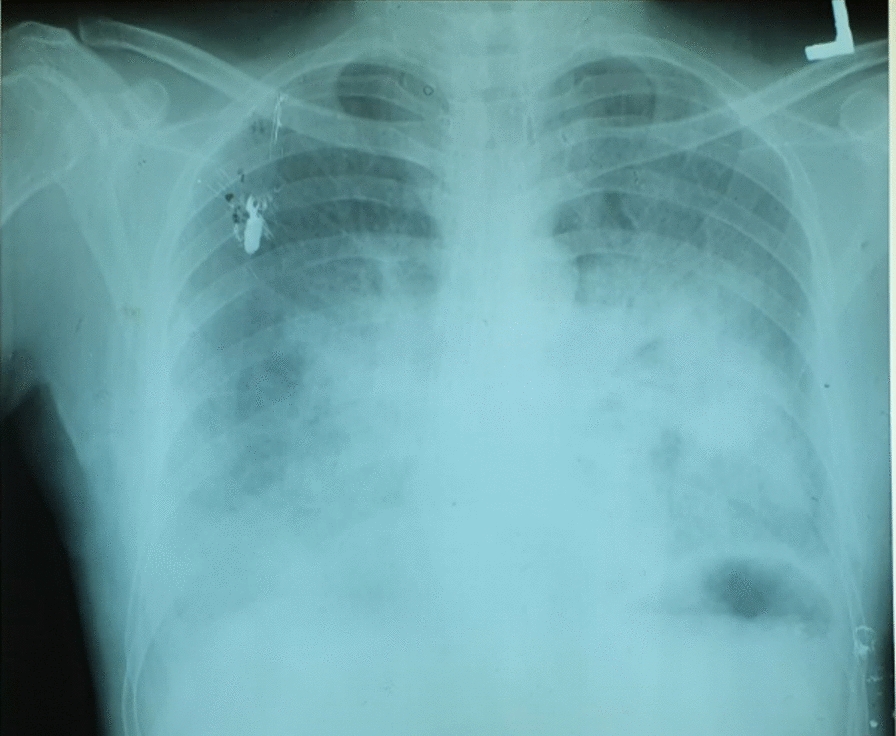
Chest radiography on the day of admission showing a normal cardiothoracic ratio and bilateral bat-wing-shaped interstitial and alveolar opacities compatible with pulmonary edema

**Fig. 2:**
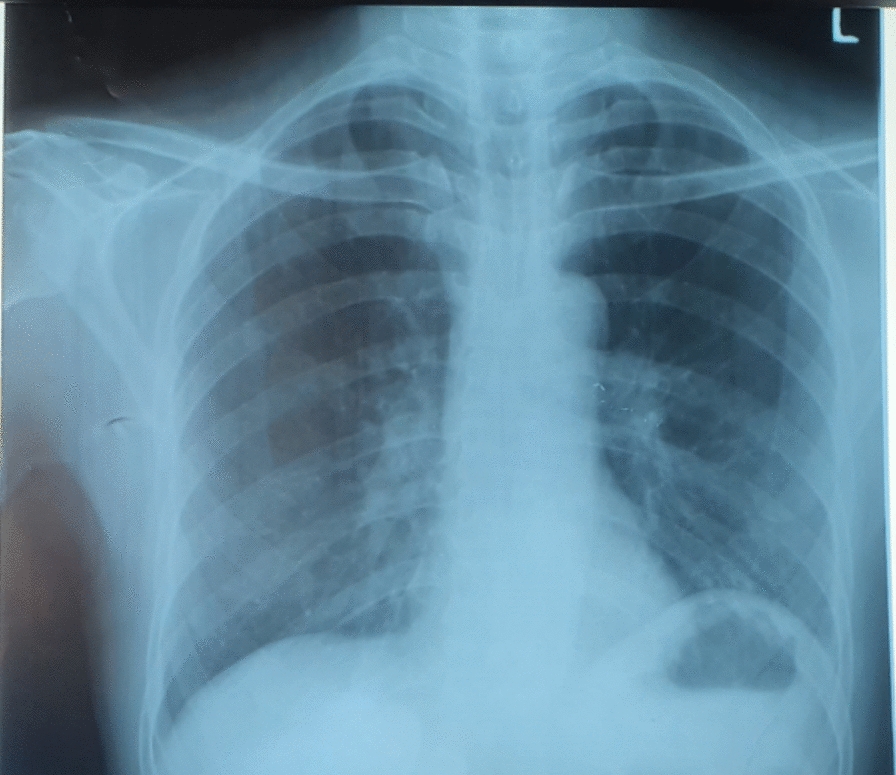
Marked improvement of pulmonary edema after improved hypoglycemic neurologic insult

A 55-year-old Eritrean male was brought to our emergency room after he became unresponsive at home. It is known that he has had diabetes for the past 25 years and was on insulin. He was noncompliant and was adjusting his insulin dose without consulting his primary physician and had had multiple admissions for hypoglycemia as a result. He came home from his daily work at 6 PM in relatively good health, except for feeling hungry owing to the daylong Ramadan fasting. He was communicating. He then took 20 IU Lente insulin and 10 IU regular insulin without breaking his fasting. After about 30 minutes, he started to have weakness and sweating. This was followed by dizziness, difficulty in concentration, and confusion. These symptoms did not improve, despite his wife trying to give him sugar. After about 2 hours he developed progressively severe shortness of breath. He was brought to our emergency room 3 hours after symptom onset. Presentation to our hospital was delayed, as the seriousness of his condition was initially overlooked because he had had similar symptoms before; in addition, there was transport unavailability on that rainy night. The patient had no personal or family history of cardiac disease. He had no history of consumption of alcohol, tobacco, or illicit drugs. He is married, living together with his spouse in Teseney town in a hut. He is a father of three and cannot read or write. He works as a welder, which is where the family income mainly comes from. He has no history of medication intake or of any intervention done for other medical conditions. Upon arrival, vitals were recorded as a blood pressure (BP) of 110/80, pulse rate (PR) of 107 beats/minute, respiratory rate (RR) of 27 breaths/minutes, temperature (T) of 33.3 °C, oxygen saturation (SpO_2_) of 37%, and random blood sugar (RBS) of 58 mg/dL. Chest exam revealed bilateral fine crepitations. There was no bronchial breath sound or wheezing, and his chest was resonant to percussion throughout. Regarding the cardiovascular system, S1 and S2 were well heard, and no murmur or gallop were appreciated. Jugular venous pressure (JVP) was not elevated, and peripheral pulses were of full volume and regular. There was no palpable enlarged organ on abdominal examination. There was no edema, pallor, or cyanosis on extremity exam. Central nervous system exam (CNS) revealed a Glasgow Coma Scale (GCS) score of 9/15 (best eye response [E] = 2, best verbal response [V] = 2, and best motor response [M] = 5), normally sized pupils reactive to light without eyeball movement abnormality, positive corneal reflex, and normotonic and normoreflexic extremity, without facial asymmetry and negative meningeal signs. With the assessment of hypoglycemic neurological insult and pulmonary edema, a peripheral intravenous line was opened in the emergency room, and 25 g dextrose 50% diluted with normal saline was administered. The patient was supported with oxygen, given via a nonrebreather mask from oxygen cylinder. Electrocardiography showed only mild tachycardia with no other remarkable changes. Chest radiography showed bilateral bat-wing-shaped interstitial and alveolar opacities compatible with pulmonary edema (Fig. 1). The cardiothoracic ratio was normal. As there were only four beds in the emergency room and no special care unit in the setup, he was transferred to the general medical ward. The patient’s consciousness improved steadily following the correction of hypoglycemia, and he attained a GCS score of 15/15 after 4 hours. His oxygen saturation also improved, and marked improvement was noticed after his GCS score reached 15/15, as evidenced by significant improvement of pulmonary edema and oxygen requirement. He was weaned off of oxygen after 24 hours of admission. Repeated chest X-ray showed a marked improvement in pulmonary edema (Fig. 2). His hemoglobin A1c was 8.6%, and his complete blood count was within normal range. Two-dimensional echocardiography performed by two general practitioners trained in echocardiography (ECHO) revealed normal intracardiac dimensions, normal regional wall motions, and normal cardiac valves, except for mild age-related aortic calcifications. Age-related diastolic impairment, with an E/A ratio of 80%, was detected. The estimated left ventricular ejection fraction was 55–60%. He was discharged, with his condition improved, on the third day of admission, with no neurologic or respiratory complaints. Insulin dosage correction and necessary advice were given with proper follow-up. Follow-up after 2 weeks revealed a fasting blood sugar of 161 mg/dL with no hypoglycemia symptoms in between, and the patient was doing his previous welding work without difficulty. Blood chemistry revealed normal renal function test. To date, over the course of a follow-up period of 6 months, he has had no complaint and has been compliant to his insulin regimen, and hemoglobin A1c has reduced to 7.2%.

## Discussion

Severe hypoglycemia can lead to severe neurological dysfunction and even death. Similarly, it can cause a neurogenic type of pulmonary edema [3, 6, 10, 14]. The presence of bilateral opacities on radiography, with a normal size heart, no evidence of left atrial hypertension, the presence of central nervous system (CNS) injury, and the absence of other common causes of acute respiratory failure or acute respiratory distress syndrome are the proposed diagnostic criteria of NPE [15]. Similarly, our patient presented with acute pulmonary edema following hypoglycemic neurologic insult, indicating that the pulmonary edema was likely a neurogenic type of NCPE.

The absence of a previous history of cardiac illness, a non-raised JVP, the absence of murmurs, a normal cardiac silhouette on radiograph, normal QRS complexes and ST segment, the absence of pathological Q-waves on electrocardiogram (ECG), and normal echocardiographic findings essentially indicate that the pulmonary edema was likely to be other than cardiac in origin in our patient. The rapid improvement of pulmonary edema only after regaining of consciousness further backs this. A similar case of NCPE owing to hypoglycemia reported that echocardiography had essentially ruled out cardiogenic pulmonary edema [14].

Previous case reports described a clinical hyperadrenergic status during hypoglycemia-induced NCPE, manifesting as tachycardia, hypertension, and dilated pupils [3–6, 14]. Despite the level of extreme hypoxia, our patient’s respiratory and pulse rates were not proportionally raised. The presence of hypothermia with these findings suggests associated hypoglycemic neurological dysfunction affecting cardiovascular, respiratory, and temperature regulation centers in our patient. A report by Andrei *et al*. presented hypoglycemic NCPE associated with progressive bradycardia, which suggested associated pontine dysfunction [14].

The RBS measured on presentation was not low enough to correlate with the hypoglycemic alteration of consciousness. Our patient showed typical hypoglycemia symptoms after taking insulin while he was fasting. The glucose we measured might be the sugar he was given on his way to our emergency by his wife rather than the true hypoglycemia that caused him neurological dysfunction at home. Similar reports where the arrival glucose level was 55 mg/dL^3^ and 60 mg/dL^5^ and was associated with hypoglycemic pulmonary edema can be found. All these cases were associated with hypoglycemic alteration of consciousness, resulting in pulmonary edema, similar to our case.

There was difficulty in differentiating from aspiration pneumonitis at first presentation, as they both develop in the setting of altered consciousness and result in respiratory distress. However, pulmonary edema in hypoglycemia and other causes of neurogenic pulmonary edema tend to develop more rapidly than aspiration pneumonia, similar to our case. The absence of vomiting or choking in the history and the bilateral fine crackles on examination indicated likely pulmonary edema. The absence of fever, focal opacities, leukocytosis, and very rapid improvement after regaining consciousness makes aspiration pneumonitis and other sepsis-induced pulmonary edema in our case very unlikely.

Our patient showed marked improvement after regaining consciousness with supportive oxygen therapy. Many episodes of hypoglycemic NCPE resolve within 24–72 hours, similar to NPE. The management measures are supportive, of which oxygen support is the main measure, and usually, improvement occurs when the central nervous insult improves. Ortega *et al*. reported a 19-year-old woman with hypoglycemic pulmonary edema that improved with oxygen who left the intensive care unit after 24 hours of admission, with normal chest X-ray and blood gases [5]. In another report by Dr. YY Mishriki, a 23-year-old man presented after hypoglycemic unconsciousness, and his pulmonary edema improved 48 hours after supportive treatments only, and his chest radiograph showed complete resolution of pulmonary edema on the third day [3].

## Conclusion

Our case report underscores noncardiogenic pulmonary edema owing to hypoglycemic neurologic insult. Different cases of hypoglycemia causing pulmonary edema have been reported, but still, medical textbooks have not included hypoglycemia among the potential causes of noncardiogenic pulmonary edema. Our case explores an atypical complication of hypoglycemic neurologic insult and adds some understanding to the existing literature that hypoglycemia can cause noncardiogenic pulmonary edema. This very unusual manifestation happened after a delayed hospital visit following hypoglycemic neurologic insult.

## Funding statement

There was no source of funding for this case report.

## Data Availability

All available information is included in the manuscript.

## Abbreviation

ECG: Electrocardiography
BP: Blood pressure
PR: Pulse rate
RR: Respiratory rate
T: Temperature
GCS: Glasgow Coma Scale
JVP: Jugular venous pressure
NCPE: Noncardiogenic pulmonary edema
NPE: Neurogenic pulmonary edema
CNS: Central nervous system
RBS: Random blood sugar
